# Prevalence of disability and associated functional limitations among older adults in Brazil

**DOI:** 10.1371/journal.pgph.0003225

**Published:** 2024-11-14

**Authors:** Rayone Moreira Costa Veloso Souto, Rafael Belo Corassa, José Veloso Souto Júnior, Otaliba Libânio Morais Neto

**Affiliations:** 1 Institute of Tropical Pathology and Public Health, Federal University of Goiás, Goiânia, Goiás, Brazil; 2 Integral Geriatric Medical Assistance–Brasília, Distrito Federal, Brasília, Brazil; African Population and Health Research Center, KENYA

## Abstract

**Introduction:**

Disabilities are a serious public health, social and human rights issue. Few studies address the relationship between disabilities and functioning among older adults. The study aimed to estimate the prevalence of disability and its’ association with comorbidities and functional limitations in Brazilian elderly individuals.

**Methods:**

Data from the National Health Survey—PNS 2019 was used. Prevalence rates with its corresponding 95% confidence intervals (CI) were estimated for variables of interest. Chi-squared tests and multiple logistic regression were conducted to investigate associations and estimate crude and adjusted odds ratios (OR) using Stata 17.0 software. The critical value (p<0.05) was considered.

**Results:**

The overall prevalence of disability was 58.3% (95% CI 57.2–59.4). Moderate/severe disabilities accounted for 24.1 (95% CI 23.1–25.1) and was high among elderly people females (27.9%, 95% CI 26.5–29.3), unemployed (28.4%, 95% CI 27.3–29.6), with an income of up to one minimum wage (30.6%, 95% CI 29.1–32.2), lower education (28.7%, 95% CI 27.5–29.9) and not married (28.5%, 95% CI 27.1–29.9*)*. Crude odds ratios of having functional limitations were 4.5 times higher among individuals with three or more comorbidities, and 32.5 times higher among those with two or more disabilities, compared to those without these conditions.

**Conclusion:**

Having a disability is an important predictor of functional limitations, especially among women, and people with lower income and education. To address this problem, public health policies such as encouraging physical activity among the elderly in Brazil should be implemented.

## Background

Disability is a serious public health, social, and human rights issue [1,2]. It leads functional limitations which negatively impact social and economic well-being [1,2]. It is estimated that over 15% of the world’s population live with some sort of disability, and nearly 200 million people suffer from some significant functional limitation [3]. The issue is expected to escalate due to the increasing burden of non-communicable diseases and aging of the population [3]. Brazilian legislation defines disability as the loss or abnormality of a psychological, physiological, or anatomical structure or function of the human body [4], such as difficulty in seeing, hearing, speaking, walking, or engaging in intellectual activities.

On the other hand, functional limitation refers to the difficulty in performing daily tasks that are essential for self-care, autonomy, and independence in social contexts [5–7]. Functional limitations are generally grouped into activities of daily living (ADL), that comprise self-care tasks such as bathing, eating, and climbing stairs independently, and instrumental activities of daily living (IADL), which refer to community life tasks, such as shopping and managing finances [8,9]. Therefore, functional capacity is a broader concept as it measures the degree of independence in performing social and self-care activities [8].

While analyses tend to focus on classifying types of disabilities [10], few studies have explored the relationship between disability and functionality [10–13]. This approach overlooks the importance of a comprehensive understanding of human functionality, including identification of both the type of disability and severity of functional limitations, to develop effective public policies [13]. In Brazil, Malta et al. [10] described disabilities by type and degree of impairment in the general Brazilian population. However, this approach does not allow a deeper understanding, and may even underestimate the impact of the problem on older individuals, since the causes of disability vary across different life stages [3,10,14]. Another Brazilian population-based study investigated the association between patterns of multimorbidity and functionality among the elderly but did not consider disability as an exposure variable [5]. Lastly, two local studies [13,15] analyzed predictors of functional limitation in the elderly; however, they did not consider disability, and the samples were limited to elderly individuals attending three primary care units [13] and one emergency hospital [15].

The study aimed to estimate the prevalence of disability and analyse the association between disability, comorbidities and functional limitations among older adults in Brazil. The study innovates by focusing on the type, co-occurrence and severity of these events among older adults. It also delves deeper into the profile of disabilities in this population, incorporating socioeconomic and demographic variables.

## Materials and methods

### Ethical declaration

The study utilized secondary, aggregate, anonymized data from the Brazilian National Health Survey—PNS 2019, publicly available on the website Brazilian Institute of Geography and Statistics. The PNS 2019 project, which originated the dataset used in this study, was approved by the National Commission for Ethics in Research (CONEP)/National Health Council (CNS) under Opinion No. 3,529,376, issued on August 23, 2019 [16].

### Study design and population

**Fig 1:**
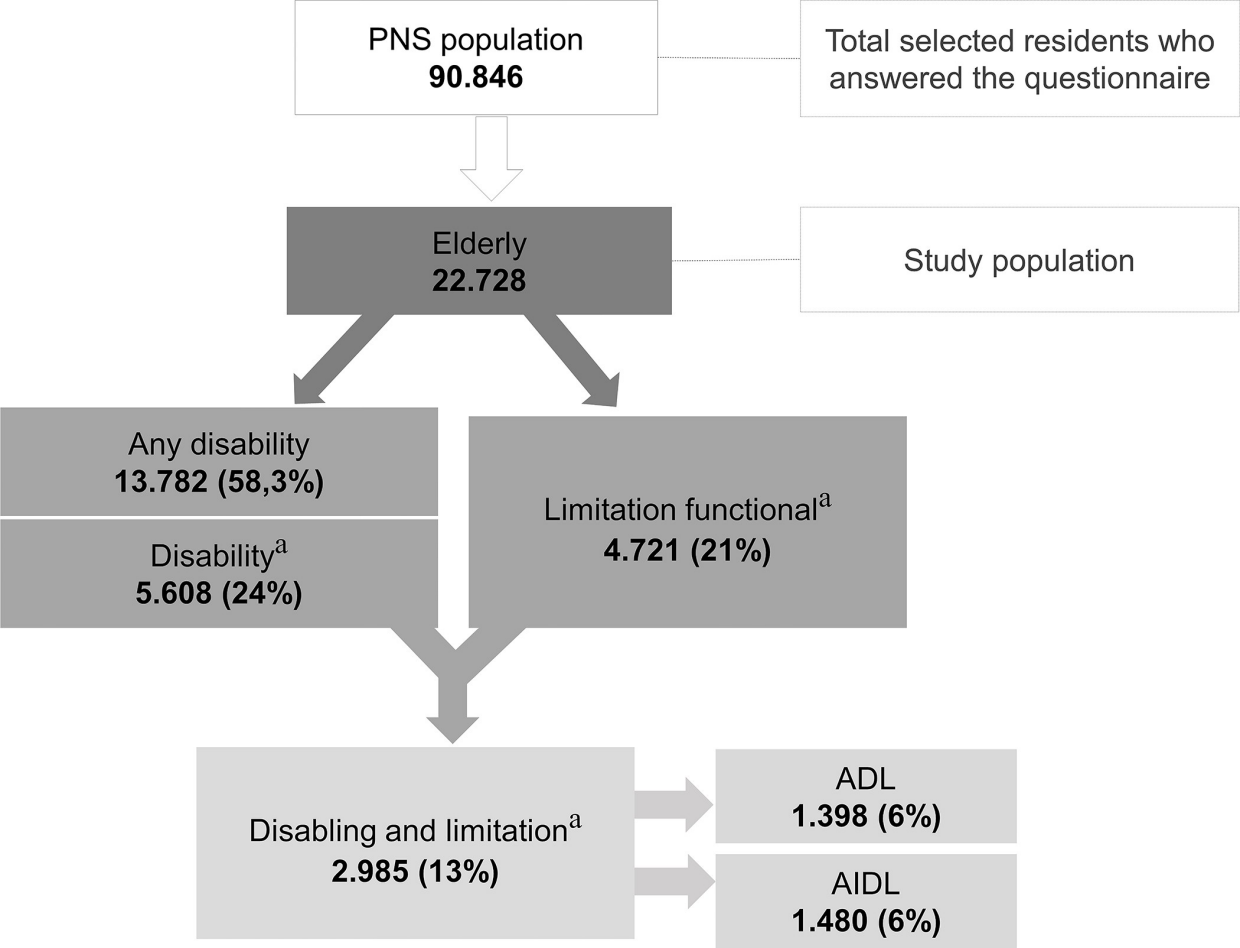
Older adults with disabilities and functional limitations based on the sample from the PNS, 2019. **Note:** ^a^Disabling includes only severe and moderate cases.

A cross-sectional study was conducted using data from the PNS 2019, collected between August 2019 and March 2020. The study population comprised the sample of elderly individuals aged 60 years or older (n = 22,728), residing in private households. Fig 1 illustrates the sample sizes of elderly individuals with disabilities and functional limitations. The complete methodology was published by Stopa et al. [16].

### Independent variables

#### Disability

Four domains of disabilities were studied: disability: visual, hearing, motor and intellectual. Disabilities were classified according to severity: none (no difficulty); mild (some difficulty); moderate (lot of difficulty); and severe (cannot do it at all). An additional category of incapacitating disability was created my merging the moderate and severe levels of disability. Disabilities were also classified according to the type (motor, visual, hearing + intellectual, or any disability) and number of disabilities (none, one, two, three of more).

#### Comorbidities

Number of comorbidities (none; one; two; three or more) considered self-reported medical diagnoses of: hypertension; diabetes; heart disease (heart attack, angina, heart failure, or other); stroke—Cerebrovascular Accident; asthma; arthritis/rheumatism; spine problems; mental illness (depression, anxiety disorder, panic disorder, schizophrenia, bipolar disorder, psychosis, OCD—Obsessive Compulsive Disorder, or other); COPD—Chronic Obstructive Pulmonary Disease; cancer; and chronic kidney disease.

#### Socio-economic and demographic characteristics

Socio-economic and demographic characteristics included: sex (male; female); race/ethnicity (black ─ brown and black; non-black ─ white, yellow, and indigenous); marital status (married; not married ─ including divorced, separated, widowed, single); income (up to 1; between 1 and 3; more than 3 minimum-salaries); employment status (not employed; employed); education level (up to completed elementary school; incomplete/completed high school; incomplete/completed higher education); residential area (rural; urban); Brazilian regions; comorbidities (none; one; two; three; and more).

### Dependent variable

#### Functional limitation

Functional limitations were classified into—limitations in [1] ADLs and [2] IADLs. ADLs include eating, bathing, dressing, using the toilet, putting on shoes, walking, and getting up; and IADLs include difficulty with shopping, managing finances, taking medication, going to the doctor, and using transportation [8]. Individuals who reported having great difficulty or being unable to perform any of these activities were considered to have functional limitations.

#### Analysis of results

General prevalence rates of disabilities were estimated according to severity (mild, moderate, and severe), type, and number of disabilities. Further analyses focused on comparing no/mild disability versus moderate/severe cases of disability, considering that these cases will significantly impair bodily functions and daily activities. Prevalences of moderate/severe disabilities were estimated according to socioeconomic and demographic characteristics, comorbidities, and region of residence of the respondents.

Bivariate analyses were conducted using Chi-squared tests (χ2). Crude odds ratios (OR) and adjusted odds ratios (AOR) were estimated using logistic regression, adjusting for potential confounding variables, considering both biological and statistical plausibility. Variables that showed significant associations (p-value < 0.05) in bivariate analysis (comorbidities, sex, employment status, income, marital status and residential area) were included. Education level was not included in adjusted analysis since it was strongly correlated to income. All analyses considered a 5% significance level and were performed using the survey module in Stata software, version 17.

## Results

**Fig 2:**
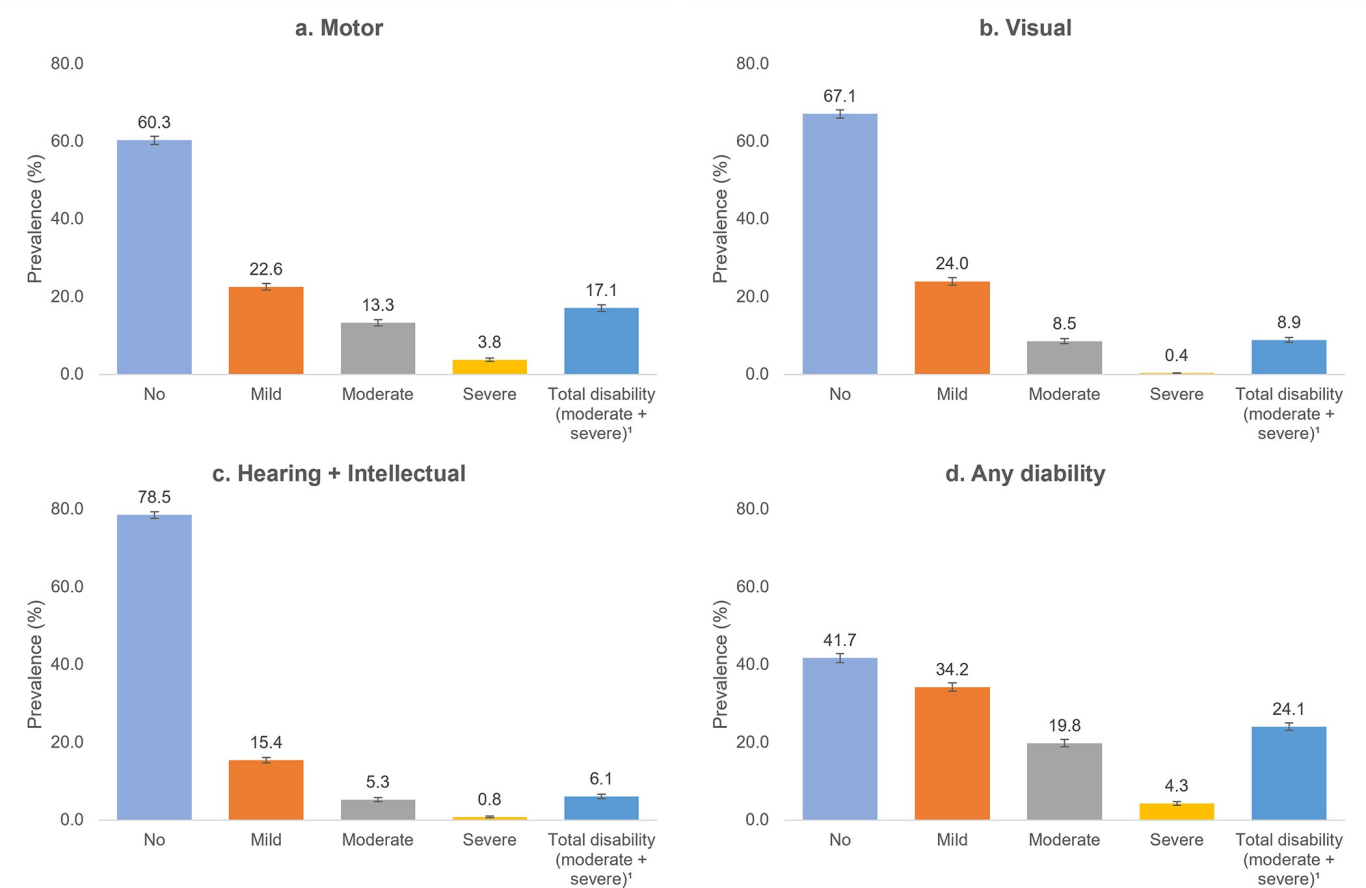
Prevalence rates of disability by type and severity among elderly individuals in Brazil. PNS, 2019. [a] Motor disability; [b] Visual disability; [c] Hearing/Intellectual disability; [d] Any disability.

The analysis included 22,728 individuals aged 60 years or older, which corresponded to 24.5% of the total sample. In Brazil, 58.3% (95% CI 57.2–59.4) of the elderly population exhibited some degree of disability, with 19.8% (95% CI 18.9–20.7) being classified as moderate and 4.3% (95% CI 3.8–4.7) as severe. Together, moderate/severe disabilities accounted for 24.1% (95% CI 23.1–25.1) of the sample. Motor (17.1%, 95% CI 16.3–18.0) and visual impairment (8.9%, 95% CI 8.3–9.6) were the most prevalent types of impairment (Fig 2).

Higher prevalence rates of moderate/severe disabilities were observed among females (27.9%; 95% CI 26.5–29.3), black individuals (26.4%, 95% CI 25.1–27.8), unemployed (28.4%, 95% CI 27.3–29.6), who earned up to one minimum-salary (30.6%; 95% CI 29.1–32.2), and with lower education level (28.7%, 95% CI 27.5–29.9). The prevalence of visual impairment was higher among residents in rural areas (11.5%, 95% CI 10.2–13.0), with an OR of 1.41 (95% CI 1.20–1.67) (Table 1).

**Table 1:** Prevalence rates of disability^a^, by type of impairment, according to socioeconomic characteristics and number of comorbidities, among elderly individuals in Brazil. PNS, 2019.

	Motor		Visual		Any disability^b^	
	n = 3855		n = 2230		n = 5608	
	% (95% CI)	OR (95% CI)	% (95% CI)	OR (95% CI)	% (95% CI)	OR (95% CI)
**Sex**						
Male	11.5 (10.5–12.5)	1	7.7 (7.0–8.5)	1	19.1 (17.9–20.4)	1
Female	21.4 (20.2–22.7)	2.10^**3**^ (1.86–2.37)	9.8 (8.9–10.8)	1.30^**2**^ (1.12–1.52)	27.9 (26.5–29.3)	1.64^**3**^ (1.47–1.82)
**Raça/Cor**						
Non-black	16.1 (14.96–17.26)	1	7.2 (6.4–8.0)	1	21.9 (20.6–23.3)	1
Black [black + brown]	18.2 (17.06–19.46)	1.16^**2**^ (1.04–1.30)	10.8 (9.9–11.8)	1.58^**3**^ (1.35–1.84)	26.4 (25.1–27.8)	1.28^**3**^ (1.16–1.41)
**Marital status**						
Married	13.3 (12.26–14.46)	1	7.0 (6.3–7.8)	1	19.8 (18.5–21.1)	1
Not married^c^	21.0 (19.76–22.27)	1.73^**3**^ (1.53–1.95)	10.9 (9.9–11.9)	1.63^**3**^ (1.40–1.90)	28.5 (27.1–29.9)	1.62^**3**^ (1.46–1.79)
**Income (minimum-salaries)**						
≤ 1	21.8 (20.4–23.3)	1	13.4 (12.2–14.6)	1	30.6 (29.1–32.2)	1
1–3	15.4 (14.2–16.6)	0.65^**3**^ (0.58–0.74)	6.5 (5.7–7.4)	0.45^**3**^ (0.38–0.53)	21.8 (20.4–23.4)	0.63^**3**^ (0.57–0.71)
≥ 3	9.2 (7.9–10.8)	0.36^**3**^ (0.30–0.44)	3.6 (2.7–4.7)	0.24^**3**^ (0.18–0.32)	12.7 (11.1–14.4)	0.33^**3**^ (0.28–0.39)
**Employment status**						
Not employed	20.7 (19.7–21.8)	1	10.2 (9.41–1.0)	1	28.4 (27.3–29.6)	1
Employed^d^	6.2 (5.1–7.5)	0.25^**3**^ (0.20–0.31)	5.0 (4.1–6.3)	0.47^**3**^ (0.37–0.60)	11.0 (9.6–12.6)	0.31^**3**^ (0.26–0.36)
**Education level**						
Up to elementary school	20.3 (19.2–21.4)	1	11.1 (10.3–12.0)	1	28.7 (27.5–29.9)	1
Complete/incomplete high.school	11.8 (10.2–13.5)	0.52^**3**^ (0.44–0.62)	4.6 (3.7–5.7)	0.39^**3**^ (0.30–0.49)	15.6 (13.9–17.5)	0.46^**3**^ (0.40–0.53)
Complete/incomplete higher.education	6.8 (5.5–8.5)	0.29^**3**^ (0.23–0.37)	2.4 (1.5–3.7)	0.19^**3**^ (0.12–0.31)	10.2 (8.5–12.2)	0.28^**3**^ (0.23–0.35)
**Area of residence**						
Rural	16.1 (14.6–17.8)	1	11.5 (10.2–13.0)	1	25.4 (23.4–27.4)	1
Urban	17.3 (16.3–18.3)	1.08 (0.95–1.25)	8.5 (7.8–9.2)	0.71^**3**^ (0.60–0.84)	23.8 (22.8–25.0)	0.92 (0.82–104)
**Comorbidities**						
None	7.6 (6.6–8.8)	1	6.1 (5.3–6.9)	1	13.2 (11.9–14.6)	1
One	11.1 (10.1–12.2)	1.52^**3**^ (1.26–1.83)	6.7 (5.9–7.6)	1.12 (0.92–1.37)	17.8 (16.5–19.2)	1.42^**3**^ (1.23–1.65)
Two	20.0 (18.3–21.9)	3.04^**3**^ (2.51–3.69)	8.7 (7.6–10.0)	1.49^**3**^ (1.21–1.83)	27.3 (25.3–29.3)	2.46^**3**^ (2.11–2.87)
Three and more	36.6 (34.1–39.2)	7.02^**3**^ (5.79–8.51)	16.7 (14.6–19.1)	3.11^**3**^ (2.51–3.85)	45.6 (42.9–48.2)	5.50^**3**^ (4.70–6.43)

Note

^1^p-value < 0.05

^2^ p-value < 0.01

^3^ p-value < 0.001.

^a^Include only moderate/severe cases of disability.

^b^Include motor, visual, hearing and intellectual disabilities.

^c^Include single, separated/divorced and widowed individuals.

^d^Includes individuals who had 1 of more jobs during the survey’s reference week.

Increasing levels of education and income were associated with decreased prevalence rates of disability. Specifically, individuals with higher education were 72% (OR = 0.28, 95% CI 0.23–0.35) less likely to have a disability, while those who earned more than three minimum salaries were 67% (OR = 0.33, 95% CI 0.28–0.39) less likely (Table 1).

Comorbidities were reported by 86.8% of respondents with disabilities, and there was a significant increase in the prevalence of disability as the number of comorbidities increased. Individuals with three or more comorbidities were 5.5 times more likely (95% CI 4.7–6.4) to having a disability compared to those without any comorbidities (Table 1).

Individuals who were not married were more likely to have a disability, being 1.73 times more likely to have motor impairment and 1.63 times more likely to have visual disability. Being employed was associated with a 75% lower chances of reporting motor disability, 53% lower chances for visual, 63% for hearing, and 95% for intellectual disability (the latter two not shown) (Table 1).

**Fig 3:**
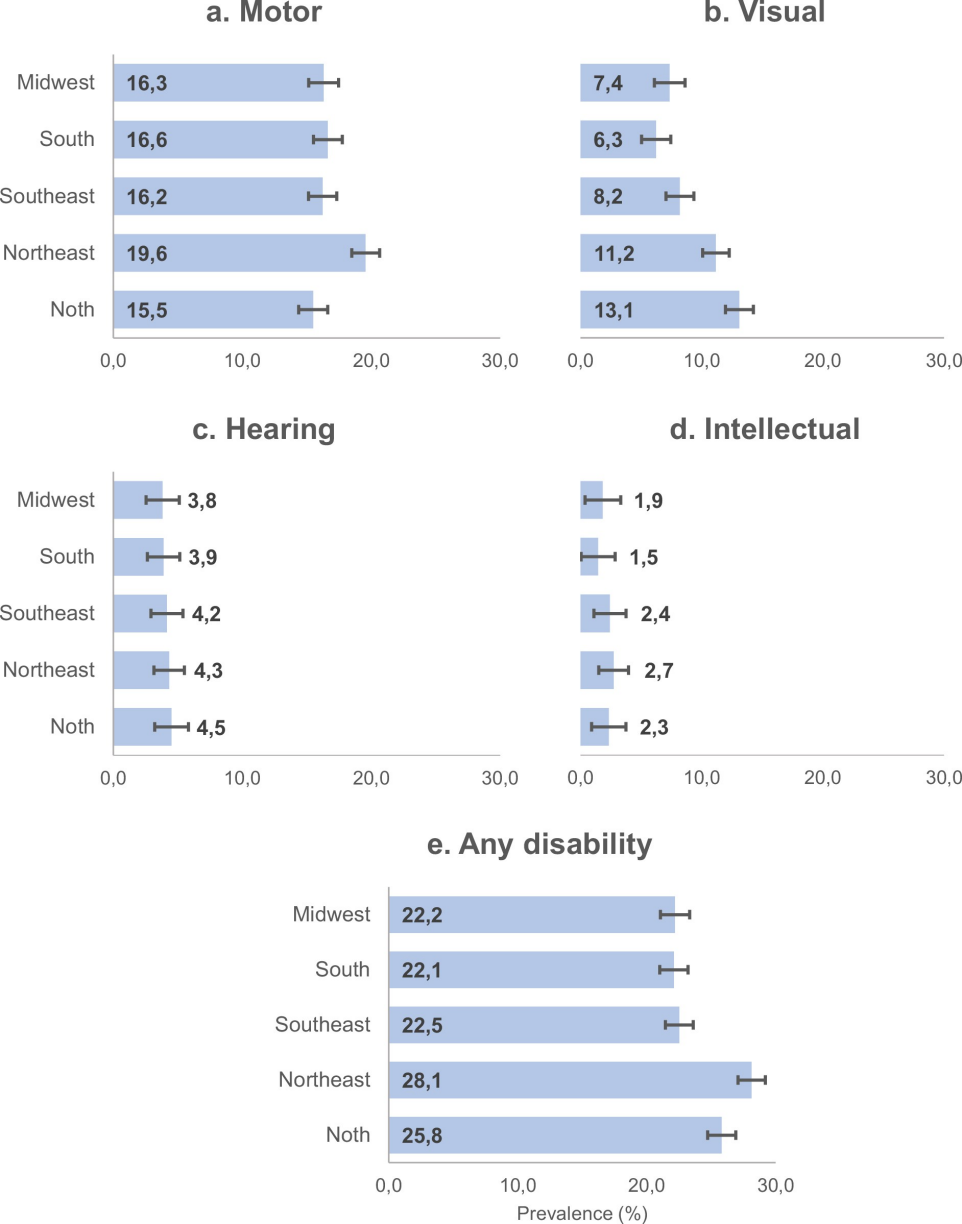
Prevalence rates of moderate/severe motor, visual, hearing and intellectual disabilities across Brazilian geographic regions, the elderly. PNS, 2019. **Nota:** ^a^Disabling includes only severe and moderate cases. ^b^Includes motor, visual, hearing and intellectual disabilities.

Analyses of prevalence rates of disability across Brazilian regions, higher rates were found in the Northeast (28.1%, 95% CI 26.6–29.8) and North (25.8%, 95% CI 23.6–28.1) regions. While higher prevalence rates of motor disabilities were observed in the Northeast (19.6%, 95% CI 18.1–21.1), higher prevalence rates of visual disabilities were observed in the North (13.1%, 95% CI 11.4–15.1) and Northeast (11.2%, 95% CI 10.2–12.3) regions (Fig 3).

The prevalence of functional limitations among individuals with disabilities was 21%. Elderly individuals with moderate/severe disabilities were 11.7 times more likely (95% CI 10.3–13.3) to report functional limitations, including limitations in ADLs (OR = 15.6, 95% CI 12.9–18.9), IADLs (OR = 11.1, 95% CI 9.8–12.6), compared to those without disabilities (Table 2).

**Table 2:** Associations between disability, comorbidities and sociodemographic characteristics and functional limitations among elderly individuals in Brazil. PNS, 2019.

	Functional limitations^a^					
	ADLs (n = 1.839)		AIDLs (n = 4.260)		Any (n = 4.721)	
	OR	(CI 95%)	OR	(CI 95%)	OR	(CI 95%)
**Disability**	** **	** **	** **	** **	** **	** **
No	1		1		1	
Yes^a^	15.58^**3**^	(12.83–18.93)	11.09^**3**^	(9.75–12.61)	11.71^**3**^	(10.34–13.25)
**Type of disability** ^**a**^						
Visual						
No	1		1		1	
Yes	4.22^**3**^	(3.38–5.27)	4.96^**3**^	(4.24–5.80)	5.19^**3**^	(4.43–6.07)
Hearing						
No	1		1		1	
Yes	2.98^**3**^	(2.21–4.02)	5.16^**3**^	(4.09–6.52)	4.62^**3**^	(3.65–5.85)
Motor						
No	1		1		1	
Yes	20.47^**3**^	(17.06–24.56)	12.75^**3**^	(11.14–14.59)	14.55^**3**^	(12.76–16.59)
Intellectual						
No	1		1		1	
Yes	20.94^**3**^	(14.84–29.54)	31.45^**3**^	(18.55–53.34)	37.18^**3**^	(20.62–67.03)
**Number of disabilities**						
None	1		1		1	
One	10.84^**3**^	(8.82–13.32)	7.82^**3**^	(6.83–8.95)	8.25^**3**^	(7.23–9.41)
Two and more	32.64^**3**^	(25.46–41.83)	28.47^**3**^	(22.27–36.39)	32.56^**3**^	(26.26–40.36)
**Comorbidities**						
None	1		1		1	
One	1.37^**3**^	(1.07–1.77)	1.35^**3**^	(1.14–1.60)	1.38^**3**^	(1.17–1.63)
Two	2.13^**3**^	(1.65–2.75)	2.41^**3**^	(2.01–2.89)	2.38^**3**^	(2.00–2.82)
Three and more	4.64^**3**^	(3.58–6.01)	4.33^**3**^	(3.61–5.19)	4.51^**3**^	(3.80–5.34)
**Sex**						
Male	1		1		1	
Female	1.37^**3**^	(1.17–1.61)	1.91^**3**^	(1.69–2.14)	1.84^**3**^	(1.64–2.06)
**Area of residence**						
Urban	1		1		1	
Rural	0.97	(0.80–1.17)	1.26^**2**^	(1.10–1.45)	1.19^**2**^	(1.05–1.36)
**Employment status**						
Employed^b^	1		1		1	
Unemployed	3.10^**3**^	(2.28–4.22)	5.91^**3**^	(4.66–7.49)	4.30^**3**^	(3.47–5.32)
**Marital status**						
Married	1		1		1	
Not married^c^	1.33^**3**^	(1.13–1.55)	1.82^**3**^	(1.62–2.03)	1.72^**3**^	(1.54–1.92)

Note

^1^p-value < 0.05

^2^ p-value < 0.01

^3^ p-value < 0.001.

^a^Includes individuals with moderate/severe disabilities/functional limitations.

^b^Includes individuals who had 1 of more jobs during the survey’s reference week.

^c^Includes single, separated/divorced and widowed individuals.

All types of disabilities were associated with increased chances of reporting functional limitations, particularly motor disabilities (OR = 14.55, 95% CI 12.76–16.59) and intellectual disabilities (OR = 37.18, 95% CI 20.62; 67.03). Elderly individuals with motor disabilities were 20.5 times more likely (95% CI 17.1–24.6) to report limitations in ADLs, and those with intellectual disabilities were 31.5 times more likely (95% CI 18.6–53.3) to report limitations in IADL, compared to those without these types of disabilities (Table 2).

The increase in the number of disabilities was associated with higher prevalence rates of functional limitations. While individuals with one type of disability were 8.3 times more likely to report functional limitations, those with two or more limitations were 32.6 times more likely (95% CI 26.3–40.4). Higher odds of functional limitations were also found among females, particularly for limitations in IADL (OR = 1.9, 95% CI 1.7–2.1), residents in rural areas (OR = 1.2, 95% CI 1.1–1.4), not married (OR = 1.7, 95% CI 1.5–1.9) and unemployed individuals (OR = 4.3, 95% CI 3.5–5.3) (Table 2).

After adjustment for confounding, having a disability was associated with 9.58 times increase in the odds of having functional limitations. The types of disabilities most strongly associated with functional limitations were motor (OR = 6.42, 95% CI 4.93–8.37) and intellectual (OR = 12.22, 95% CI 6.91–21.64) disabilities (Table 3).

**Table 3:** Multiple logistic regression on the association between disability^a^ and functional limitations^b^ among elderly individuals in Brazil. PNS, 2019.

	No adjustment		Model 1		Model 2		Model 3	
			Comorbidities		Model 1 + Sex and Employment		Model 1 + 2 + Socioeconomics	
	OR	(95% CI)	AOR	(95% CI)	AOR	(95% CI)	AOR	(95% CI)
**Disability**								
No	1		1		1		1	
Yes	11.71^3^	(10.34–13.25)	11.08^3^	(9.77–12.56)	9.94^3^	(8.75–11.28)	9.58^3^	(8.44–10.89)
**Type of disability**^**b**^								
Visual								
No	1		1		1		1	
Yes	5.19^3^	(4.43–6.07)	1.89^3^	(1.51–2.37)	1.93^3^	(1.53–2.43)	1.82^3^	(1.44–2.30)
Hearing								
No	1		1		1.00		1	
Yes	4.62^3^	(3.65–5.85)	2.01^3^	(1.53–2.63)	2.04^3^	(1.55–2.69)	2.04^3^	(1.55–2.68)
Motor								
No	1		1		1		1	
Yes	14.55^3^	(12.76–16.59)	6.86^3^	(5.28–8.91)	6.41^3^	(4.92–8.34)	6.42^3^	(4.93–8.37)
Intellectual								
No	1		1		1		1	
Yes	37.18^3^	(20.62–67.03)	12.71^3^	(7.03–22.97)	11.85^3^	(6.64–21.14)	12.22^3^	(6.91–21.64)

Note

^1^p-value < 0.05

^2^ p-value < 0.01

^3^ p-value < 0.001.

^**a**^Include cases of moderate/severe disability/functional limitation.

^**b**^Included adjustment for all types of disabilities (visual, hearing, motor, and intellectual).

**OR:** Crude Odds Ratio; **AOR:** Adjusted Odds Ratio.

**Model 1:** Adjusted by comorbidities; **Model 2:** Adjusted by comorbidities, sex and employment status; **Model 3:** Adjusted by comorbidities, sex, employment status, marital status, income and area of residence.

## Discussion

This study described the prevalence rates of disabilities and examined their relationship with comorbidities and functional limitations using representative data from the elderly population in Brazil. Disability was an important predictor of functional limitations, especially among elderly women and those with lower socioeconomic conditions, such as lower education and income levels. It was also confirmed that increased frailty, characterized by the presence and severity of disabilities and comorbidities, increased the likelihood of reporting functional limitation.

### Disability and functional limitation

The study indicated that disability is strongly associated with functional limitations among elderly individuals, corroborating the findings of a previous study [10]. The prevalence of disabilities among elderly individuals in Brazil (58.3%) was also higher than the global estimate (43.4%) [3]. Furthermore, the prevalence of severe/moderate disabilities (24%) exceeded the prevalence rates found in the 2013 National Health Survey (18.2%) [10]. The growing prevalence of disabilities is a cause for concern, especially in light of the rapid aging of the Brazilian population [17,18] and the increasing burden of chronic diseases [14,17,19].

The aging process is the main contributor to conditions that lead to frailty in older adults [20–22]. Aging is characterized by a gradual loss of functional reserve and progressive changes that increase physical and cognitive frailty [23]. This process, along with sedentary habits, inadequate nutrition and social and environmental vulnerabilities, predispose elderly individuals to chronic diseases and other health conditions that can lead to decline in functionality and multiple disabilities [7,17,22–25]. The main biological changes result from oxidative stress and chronic inflammation, which cause progressive molecular and cellular damage [23,26]. In this context, social determinants of health have a significant influence on the aging process and the health conditions of individuals, since they will interfere in factors such as income, education, housing conditions, access to food, among others [27].

The highest prevalence of motor disability, as observed in another study [10], is linked to the deterioration of the musculoskeletal system, which is most affected by aging. Changes such as reduction of type 2 muscle fibres and reduction of myofibrils, resulting from chronic inflammation as well as genetic and autoimmune alterations lead to a reduction in physical aptitude, with a decrease in muscle volume, strength, and function, in a process known as sarcopenia [28]. Muscle atrophy begins at age 40, with a loss of 1% per year, and accelerates dramatically from 80 years old onwards [29]. In addition, there is an observed accumulation of fat, especially in visceral structures, which leads to a reduction in strength [30,31] and a systemic increase in cytokines and chemokines [32,33]. These factors contribute to chronic inflammation and insulin resistance, the main processes involved in the onset of cardiovascular diseases and diabetes [34].

These changes in the musculoskeletal system are also the main factors responsible for mental disabilities and functional limitations, particularly in daily life activities [35,36]. Muscle atrophy restricts mobility, reduces hand grip strength and gait, causes limb atrophy due to disuse, leads to bone thinning, weight loss, fatigue, and exhaustion, leading to a frailty phenotype [17,22–24,37–39]. As a result, there are higher rates of falls, osteoporosis, chronic diseases [24,38], care dependency, depression, dementia, and death [40–42].

Our research highlights that, second only to intellectual disability, motor disability presents a significant limitation. This finding aligns with the results of other studies [10,42]. Interestingly, cognitive function is also compromised by the reduction in muscle mass [23]. This is particularly evident in cases of dementia of vascular aetiology, also known as Vascular Cognitive Impairment (VCI) [38,42]. VCI is associated with lower survival rates, increased disability, and higher costs compared to Alzheimer’s disease [24,43].

### Comorbidities and functional limitations

Our study has found that the presence of comorbidities is also linked to a higher occurrence of functional limitations, as seen in other studies [7,11,13,15,44–46]. However, the impact on old age is determined not only by the presence of comorbidities but also by the presence and interaction of specific diseases [47]. Tinetti et al. [48] observed that some diseases tend to occur together, while others act synergically, such as depression, which exacerbates heart failure, osteoarthritis, and cognition. Thus, certain patterns of chronic diseases tend to produce more limitations. Patterns of mental, motor, and cardiopulmonary impairment are most associated with limitations in ADL and IADL [5].

For instance, in musculoskeletal diseases, such as arthritis and rheumatism, the decline in functionality mainly results from chronic pain and its consequences, such as sleep deprivation, psychological suffering, and depression [49], which affect physical conditions, functionality, and the use of health services [50]. Alternatively, cardiopulmonary diseases, such as asthma and heart diseases, have a greater potential for IADL limitations due to symptoms of dyspnoea, respiratory discomfort, and fatigue [51], which prevent the individual from performing more complex activities, such as shopping. In patients with chronic obstructive pulmonary disease (COPD), there is also a loss of muscle mass and fatigue due to physical inactivity [51], which consequently compromises the capacity to perform simple activities such as bathing.

### Socioeconomic and demographic factors

Socioeconomic factors, including education and income, were found to be correlated with disability and functional limitations, which is consistent with previous studies [13,52–54]. These factors contribute to precarious health conditions, potentially accelerating functional decline [13]. Furthermore, individuals with fewer economic resources encounter greater challenges to access quality healthcare services. This is especially true for people with disabilities, who face additional barriers in accessing rehabilitation services, education, employment, transportation, and information [3]. Conversely, higher levels of education empower individuals to make informed decisions about healthier lifestyles, including dietary choices and physical activity, and reduces exposure to physically demanding occupations and unhealthy working conditions [55].

The Northeast region of Brazil, which if characterized by poorer socioeconomic conditions, exhibited the highest prevalence of severe disabilities among older adults, a pattern consistent with findings from Neri [56]. Furthermore, there was a higher prevalence of disability among Black individuals, a disparity that can be attributed to these unfavourable socioeconomic conditions [57], as well as the inequality of access to health services, and factors such as racism, stigma and discrimination against people with disabilities and in old age [58].

Employment status demonstrated an association with disability and functional limitations, as in previous research [13,59,60]. Engaging in work appears to mitigate the decline in physical capability [61], possibly by maintaining mobility, muscle strength, and autonomy while strengthening the individual’s social support network. Employment also contributes to the financial well-being of older adults by covering additional expenses such as food and medication [60,62]. However, employment status, considering the Brazilian socioeconomic and demographic reality, must be interpreted with cautions, considering the exposure to dangerous types of work and the inequality of public social and social security policies for the elderly. In addition, working in old age may reflect the need to continue working in order to survive [63].

### Female sex

The prevalence of disabilities and the degree of dependency were higher among women, consistent with findings from other studies [15,52,53,55,64–66]. Women are more likely to have chronic diseases [11,15] and experience greater frailty than men [7,67–70]. They also report worse mental health conditions [71]. Women’s longer lifespan [13] increases their exposure to chronic diseases and associated outcomes. However, a study on the incidence of comorbidities by gender found no statistically significant difference in incidence between men and women [72].

### Marital status

Regarding marital status, our study found that being married significantly reduced the likelihood of having a disability, as seen in other studies [11,13,71] and functional limitations [7,13,15,64]. Long-standing marital cohabitation allows for the sharing of leisure-time activities, financial resources, and investments more than it would be observed with other family members or friends [73]. It also strengthens mutual bonds and support, which are essential to minimize stress and to encourage positive lifestyle changes [73], promoting better health. Conversely, marital breakup or widowhood can negatively impact the individual’s functional capacity [73].

### Main strategies for functioning

The functionality-focused approach is grounded in the paradigm of active or successful aging [3,17,56], which emphasizes the engagement with life and the maintenance of functional abilities or the adaptation to age-related losses, aiming for satisfactory levels of independence [74,75], even among individuals with disabilities and comorbidities.

Significant research has shown that physical aptitude and low-calorie diets can extend human longevity [24]. Physical activity emerges as the most effective strategy for preventing, minimizing, or reversing most physical, social, psychological [62,76,77] and mental [78], declines commonly associated with aging. Physical activity is consistently associated with significant improvements in health conditions such as stress, obesity, diabetes control, and coronary diseases [62,77], as well as cognitive and motor functions [77,79], making it the most important factor for increasing functional capacity in older adults [80].

Regarding nutrition, research has shown that caloric restriction leads to mutations in insulin signalling pathways, promoting improvements in sarcopenia, cardiovascular disease, diabetes, Alzheimer’s disease, and cancer [24,81]. Additionally, a nutritious and balanced diet contributes to overall health improvement and can intervene at all three levels of chronic disease prevention [3].

### Study limitations

The study’s limitations include the potential for recall bias due to self-reported measurements, which is inherent to the study design [5]. Given that this is a cross-sectional study, causal relationships between disabilities and functional limitations cannot be confirmed. Nonetheless, the PNS is a nationally representative study with a robust sample, allowing for various approaches and comparisons with other international studies.

## Conclusion

The study revealed that disability is strongly associated with functional limitations, especially among women and more vulnerable elderly individuals. Additionally, the high burden and relationship between comorbidities, disabilities, and functional limitations, indicate that the Brazilian population is experiencing an unhealthy aging process. This scenario highlights that the healthcare system is failing to address the rising trend of chronic diseases [82], which are the main causes of these health issues [3,5,14].

By addressing the relationship between disability, comorbidities, and functional limitations, considering severity and the multiple socioeconomic and demographic factors, the study extends and deepens previous knowledge [5,10,13], incorporating functionality as a key component in the health assessment of the elderly individuals [13,83], in a robust sample. Future studies should further investigate lifestyles and risk factors for chronic diseases and their association with functional decline, aiming to guide public policies for timely intervention at all three levels of prevention.

The effectiveness of public policies relies on interventions focused on maintaining and recovering the functionality of elderly individuals [84]. To achieve this, it is essential to intervene in social determinants that impact lifestyle and quality of life, with an emphasis on promoting physical activities and healthy eating as a public policy, and not just an individual choice. Additionally, it is necessary to address barriers and attitudes influencing of disability [3], such as healthy environments and mobility, and ensure access to qualified healthcare services, especially rehabilitation.
